# Agricultural azole fungicides induce cross-resistance to medical antifungals in *Candida tropicalis*: a One Health perspective on antifungal resistance

**DOI:** 10.1080/21501203.2026.2657142

**Published:** 2026-04-23

**Authors:** Shuaihu Li, Tianren Hu, Chao Li, Tianchen Tan, Haiqing Chu, Guanghua Huang

**Affiliations:** a Shanghai Institute of Infectious Disease and Biosecurity, State Key Laboratory of Genetics and Development of Complex Phenotypes, School of Life Sciences, Department of Infectious Diseases, Huashan Hospital, Fudan University, Shanghai, China; b Department of Respiratory and Critical Care Medicine, Shanghai Pulmonary Hospital, School of Medicine, Tongji University, Shanghai, China; c Shanghai Key Laboratory of Tuberculosis, Shanghai Pulmonary Hospital, School of Medicine, Tongji University, Shanghai, China; d College of Fisheries and Life Science, Shanghai Ocean University, Shanghai, China

**Keywords:** *Candida tropicalis*, agricultural azoles, cross-resistance, experimental evolution, One Health

## Abstract

The human opportunistic fungal pathogen *Candida tropicalis* is widely distributed in natural environments. In recent decades, the prevalence of azole-resistant isolates has increased markedly in the Asia-Pacific and Latin America. This epidemiological change could be associated with the extensive application of azole fungicides in modern agriculture. In this study, we demonstrate that exposure to four widely used agricultural azoles (difenoconazole, diniconazole, flutriafol, and hexaconazole) induces cross-resistance to medical azoles in an initially susceptible *C. tropicalis* strain. The evolved azole-resistant strains exhibited altered cellular and colony morphologies, along with changes in growth rates. Genomic analyses revealed that all evolved strains were aneuploid, with either partial chromosomal segment amplifications or extra chromosomes. Global transcriptomic analysis showed that many azole resistance-associated genes located within these amplified regions were upregulated, potentially contributing to resistance against both clinical and agricultural azoles. These findings highlight the potential role of agricultural azoles as the environmental drivers of antifungal resistance in the medically important fungal pathogen *C. tropicalis*, underscoring the importance of a One Health approach to antifungal stewardship.

## GRAPHICAL ABSTRACT

**Figure:**
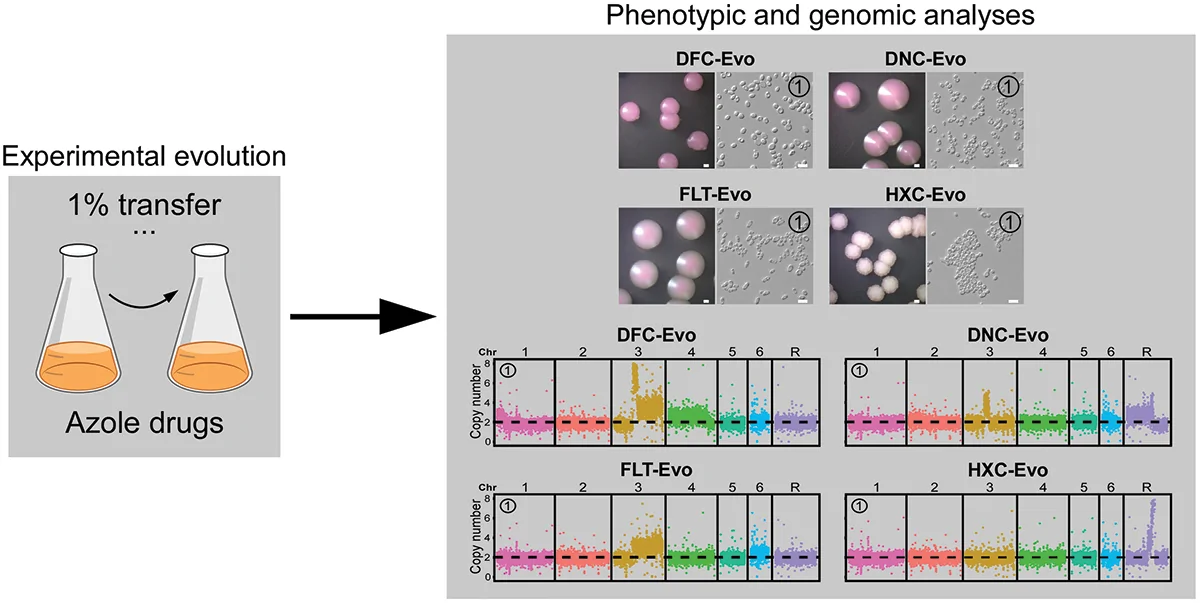

## 1. Introduction

*Candida tropicalis* is one of the most prevalent yeast pathogens in humans, capable of causing both superficial and life-threatening systemic infections (Zuza-Alves et al. 2017). Bloodstream infections caused by this fungal pathogen are associated with high mortality rates (Barac et al. 2020). Unlike *Candida albicans* and *Candida glabrata*, which primarily colonize or infect human hosts, *C. tropicalis* is both a commensal microbe and an environmental generalist (Meng et al. 2025), widely distributed in diverse habitats, such as wastewater, beaches, soil, fruits, vegetables, and trees – particularly in tropical and subtropical regions (Vogel et al. 2007; Rehman and Anjum 2011; Hallen-Adams and Suhr 2017; Aljohani et al. 2018; Chen et al. 2023). It also demonstrates high osmotolerance and can survive under elevated salt concentrations (Mackenzie 1971; Zuza-Alves et al. 2016).

Over the past two decades, azole-resistant *C. tropicalis* isolates have become increasingly common in clinical settings, especially in the Asia-Pacific and Latin America (Yang et al. 2023; Lass-Flörl et al. 2024). For instance, fluconazole resistance in China rose dramatically from 5.7% in 2009 to 31.8% in 2018 (Wang et al. 2021). The underlying drivers of this shift remain unclear. Several studies have reported azole-resistant isolates from both antifungal-exposed patients and environmental sources (Fan et al. 2017; Pfaller et al. 2022; Fan et al. 2023; Bastos et al. 2025). Notably, a dominant azole-resistant clone, MLST clade 4, has been identified in both clinical and environmental samples (Lo et al. 2017), with isolates in this clade harboring characteristic hotspot mutations in the lanosterol 14α-demethylase-encoding gene *ERG11* (Tseng et al. 2022). These findings suggest potential transmission between environmental and clinical reservoirs, facilitating the spread of azole-resistant *C. tropicalis*.

In the case of the filamentous fungal pathogen *Aspergillus fumigatus*, the overuse of agricultural azoles is a well-established driver of resistance (Badali et al. 2013; Alvarez-Moreno et al. 2017; Cao et al. 2021). Environmental exposure to these fungicides selects for resistant strains, as both agricultural and medical azoles target the same enzyme, lanosterol 14α-demethylase, encoded by *ERG11* (also known as *cyp51A* in filamentous fungi). Given the widespread environmental presence of *C. tropicalis*, a similar resistance mechanism may be at play in this species. In a recent study, we showed that exposure to the agricultural triazole tebuconazole (TBZ) induces cross-resistance to medical azoles in *C. tropicalis*, likely via genomic instability, characterized by elevated rates of chromosomal and genetic alterations (Hu et al. 2025b).

In this study, we aimed to investigate whether cross-resistance induced by agricultural fungicides is a broader phenomenon in *C. tropicalis*. Using an experimental evolution approach, we exposed an azole-susceptible strain to four widely used agricultural azoles—difenoconazole (DFC), diniconazole (DNC), flutriafol (FLT), and hexaconazole (HXC). All four fungicides successfully induced cross-resistance to medical azoles, accompanied by the emergence of aneuploidy (a cell with one or more extra or missing chromosomes). The evolved strains displayed substantial phenotypic diversity, including changes in morphology and growth rates. Our results suggest that agricultural azoles may act as environmental drivers of antifungal resistance in *C. tropicalis*, reinforcing the need for a One Health framework in antifungal resistance surveillance and management.

## 2. Materials and methods

### 2.1. Strains and cultural conditions

*C*. *tropicalis* strains (Table S1) were routinely cultured in rich medium (YPD, 20 g/L peptone, 10 g/L yeast extract, 20 g/L glucose). For morphological assays, solid YPD medium (2% agar) supplemented with 5 μg/mL phloxine B dye was used to assess changes in ploidy and colony morphology. RPMI 1640 medium was used for antifungal susceptibility assays. For experimental evolution assays, RPMI 1640 medium was supplemented with 2% (w/v) glucose. Yeast extract was purchased from Thermo Fisher Scientific (catalog no. 211930, USA); peptone and agar from BD Biosciences (catalog nos. 211677 and 214010, USA); and glucose and phloxine B from Sigma-Aldrich (catalog nos. G8270 and P2759, USA). RPMI 1640 medium was purchased from Gibco (catalog no. 31800, USA).

### 2.2. In vitro *antifungal susceptibility assays*

Antifungal susceptibility testing was performed according to the Clinical and Laboratory Standards Institute-M27 (CLSI-M27) guidelines. Antifungal agents were obtained from Sigma-Aldrich (St. Louis, MO, USA), and stock solutions of the 14 drugs used in this study were prepared at the following concentrations: DFC (16 mg/mL in DMSO), DNC (16 mg/mL in DMSO), FLT (16 mg/mL in DMSO), HXC (32 mg/mL in DMSO), FLC (16 mg/mL in DMSO), ITC (4 mg/mL in DMSO), KEC (8 mg/mL in DMSO), POS (8 mg/mL in DMSO), VRC (4 mg/mL in DMSO), 5-FC (8 mg/mL in ddH_2_O), AFG (8 mg/mL in DMSO), AmB (8 mg/mL in DMSO), CAS (8 mg/mL in DMSO), and MFG (16 mg/mL in DMSO). The working concentration range for all antifungals was 0.0625 to 64 μg/mL.

The drug-evolved strains and the progenitor strain C23 were cultured on YPD medium at 30 °C for 3 d. Approximately 1 × 10^3^ cells were then suspended in RPMI 1640 medium containing two-fold serial dilutions of each drug and incubated at 35 °C for 24 h. Three independent biological replicates were performed. Minimum Inhibitory Concentration (MIC) values were defined as the lowest drug concentration that inhibited ≥ 50% growth relative to the drug-free control.

### 2.3. Experimental evolution assays

A single colony of the progenitor strain C23 was cultured in RPMI 1640 medium containing 2% glucose at 30 °C for 24 h. Approximately 1 × 10^6^ cells were inoculated into 20 mL of fresh medium supplemented with an agricultural azole at 1× MIC [1 μg/mL for difenoconazole (DFC), 0.0625 μg/mL for diniconazole (DNC), 0.25 μg/mL for flutriafol (FLT), or 0.125 μg/mL for hexaconazole (HXC)] and incubated at 30 °C for 2 d until OD_600_ ≥ 5.0. Subsequently, 0.5 mL of culture was transferred into 19.5 mL of fresh medium containing the same azole at 2× MIC and incubated under the same conditions until OD_600_ ≥ 5.0. This passaging procedure was repeated seven times, gradually increasing the azole concentration until reaching 64× MIC. Fungal cells were then plated onto YPD agar containing the same azole at 128× MIC and incubated at 30 °C. Three biological repeats were performed for each azole treatment.

### 2.4. Morphological analysis

For morphological and phenotypic assays, *C. tropicalis* cells were grown overnight in a YPD liquid medium at 30 °C with shaking at 220 r/min. Fungal cells were harvested, washed, and plated on YPD agar containing 5 μg/mL phloxine B followed by incubation at 30 °C for 3 d. Colony morphology was documented using a stereoscopic microscope camera system (NSZ-810 + Dig1600, Ningbo Yongxin, China), and cellular morphology was examined with a Leica DM2500 optical microscope.

### 2.5. Growth curve experiment

*C*. *tropicalis* strain was streaked onto YPD agar plates and incubated at 30 °C for 3 d. After incubation, cells from a single colony were inoculated into liquid YPD medium and grown overnight at 30 °C with shaking (220 r/min). Fungal cells were then washed twice with ddH_2_O and re-inoculated into fresh YPD medium at a final concentration of 1 × 10^6^ cells/mL. Cultures were incubated at 30 °C with shaking at 220 r/min. Three biological replicates were performed. During the lag and exponential phases, cells were collected every 1.5 h. During the stationary phase, samples were collected every 2 h. Optical density (OD) was measured, and growth curves were fitted using the DMFit program.

### 2.6. Flow cytometry assays

Flow cytometry was performed to determine the ploidy of azole-evolved strains and the progenitor strain C23 as described previously (Du et al. 2018; Ji et al. 2025). Briefly, strains were streaked onto YPD agar and incubated at 30 °C for 3 d. Single colonies were inoculated into liquid YPD medium and grown overnight at 30 °C to stationary phase. Cultures were diluted into a 3-mL fresh YPD medium to an initial OD_600_ of 0.5 and grown at 30 °C, 220 r/min until OD_600_ reached ~1.8. Then, 1 mL of culture was collected, washed with ddH_2_O, resuspended in 300 μL ddH_2_O, mixed with 700 μL ethanol, and incubated at room temperature for 2 h. Cells were washed with 1× TE buffer (10 mmol/L Tris, 1 mmol/L EDTA, pH 8.0), treated with RNase A (0.3 mg/mL) at 37 °C for 2 h, and then with protease K (0.03 mg/mL) at 50 °C for 2 h. Finally, cells were washed with 1× TE, resuspended in 1× PBS, and stained with propidium iodide (PI, 25 μg/mL) for at least 1 h before DNA content analysis by flow cytometry. The progenitor strain C23 served as a diploid reference strain, with a peak at approximately 200 in its flow cytometry profile. In contrast, the progeny cells exhibited distinct DNA content peaks.

### 2.7. DNA extraction and whole-genome analysis

A single colony of each *C. tropicalis* strain was streaked onto YPD agar and incubated at 30 °C for 3 d. Genomic DNA was extracted using the TIANamp Yeast DNA Kit (Cat. No. DP307-02, Tiangen, China) following the manufacturer’s instructions. Library construction, whole-genome resequencing, and raw data processing were conducted by BGI (Wuhan, China). All clean sequencing data have been deposited in the NCBI Sequence Reads Archive (SRA) database under accession number PRJNA1232229.

Variant calling and copy number variation (CNV) analyses were performed as described previously (Gong et al. 2020). In brief, clean reads from each strain were aligned to the *C. tropicalis* reference genome MYA-3404 (NCBI accession: GCA_013177555.1) using BWA v0.7.17 (mem algorithm). Variant calling was performed using SAMTools v1.12, Picard Tools v1.56 (http://picard.source-forge.net), and the Genome Analysis Toolkit (GATK v3.4). For analysis of genome-wide copy number distribution, the average read depth was calculated in 1000-bp non-overlapping windows. The Splint script was used to correct read depth bias, such as the “smiley pattern”. To determine the degree of CNV, the relative CNV value for each 1000-bp non-overlapping window was normalized to the median of all windows. Then, genome-wide CNV distributions were visualized with the R package ggplot2. Similarly, gene-level copy numbers were determined using the “SAMtools depth” command across annotated coding sequences.

### 2.8. RNA extraction and RNA-seq analysis

*C*. *tropicalis* cells were grown in YPD medium at 30 °C for 24 h with shaking at 220 r/min. Then, total RNA was extracted using the GeneJET RNA Purification Kit (K0732, Thermo Fisher Scientific, USA) following the manufacturer’s protocol. Three biological replicates were performed for each condition.

RNA sequencing was performed on an Illumina NovaSeq 6000 platform (Berry Genomics Co, Beijing). Clean reads were aligned to the *C. tropicalis* reference genome MYA-3404 (NCBI accession: GCA_013177555.1) using HiSat2 v2.0.5. Transcript abundance was quantified with StringTie v1.3.3b, and differential expression analysis was conducted using the DESeq2 R package. Genes with a fold change ≥ 2 and a false discovery rate (FDR) ≤ 0.01 were considered significantly differentially expressed. Expression levels of the progenitor strain C23 were used as the baseline reference. Raw RNA-seq data have been deposited in the SRA database under the accession number PRJNA1232229 (SRR32584188-SRR32584226) and are publicly accessible without restriction.

## 3. Results

### 3.1. *Experimental evolution of azole-resistant* C. tropicalis *strains*

To investigate the potential role of agricultural azole fungicides in driving antifungal resistance in *C. tropicalis*, we conducted an experimental evolution study (Figure 1a). The azole-susceptible *C. tropicalis* strain C23 was inoculated into RPMI 1640 medium supplemented with 2% glucose and subjected to serial passaging in progressively increasing concentrations of four commonly used agricultural azole fungicides: difenoconazole (DFC), diniconazole (DNC), flutriafol (FLT), and hexaconazole (HXC). Once the *C. tropicalis* populations adapted to grow in medium containing concentrations up to 64× the minimum inhibitory concentration (MIC) of each azole, an aliquot of cells was plated onto YPD agar containing 128× MIC of the corresponding fungicide. Plates were incubated at 30 °C for 3 to 5 d. From each evolution condition, 10 single colonies were randomly selected from each of the three biological replicates for antifungal susceptibility testing. As shown in Figure 1b, the evolved strains exhibited a broad range of elevated MICs to the corresponding azoles. From each group, three evolved strains with the highest MICs were selected for further experiments (Table S1).

**Figure 1.**
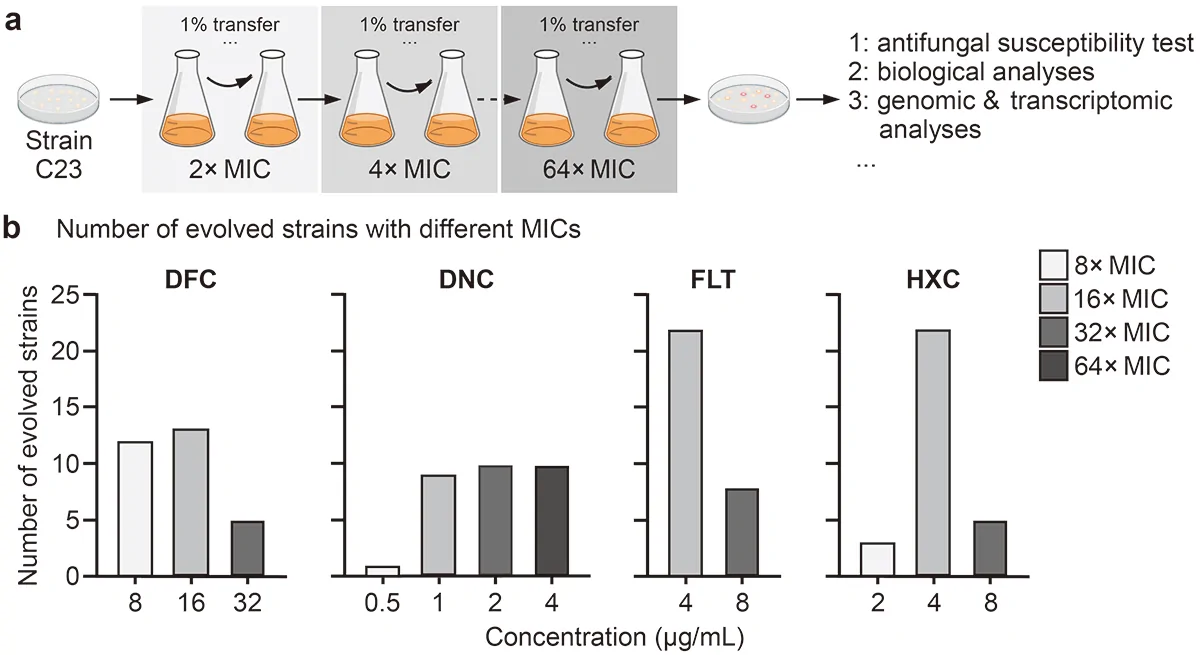
Experimental evolution of *Candida tropicalis* resistant strains to agricultural azoles. (a) Schematic of the *in vitro* experimental evolution strategy. The strains were serially passaged in media with gradually increasing drug concentrations, and the resulting evolved cells were harvested for subsequent experiments. (b) Number of evolved *C. tropicalis* strains exhibiting varying levels of susceptibility to agricultural azoles. From the final YPD agar plates in panel A, 30 single colonies were randomly selected and subject to antifungal susceptibility testing. The x-axis shows azole concentration and the y-axis indicates the number of evolved isolates.

### 3.2. *Evolved* C. tropicalis *strains exhibit cross-resistance to medical azoles*

To determine whether the evolved strains also developed cross-resistance to other agricultural fungicides and clinically used azoles, we performed comprehensive antifungal susceptibility assays. As shown in Table 1, all 12 evolved *C. tropicalis* strains, originating from exposure to DFC, DNC, FLT, and HXC, exhibited significantly reduced susceptibility to the three other agricultural azoles, as well as one or more medical azoles, when compared with the progenitor strain C23. Notably, strains evolved under DFC and FLT pressure showed markedly elevated MICs for fluconazole (FLC), itraconazole (ITC), and voriconazole (VRC). Similarly, DNC- and HXC-evolved strains exhibited high MICs for FLC, ITC, posaconazole (POS), and VRC. Although all evolved strains retained relatively low MICs to ketoconazole (KEC), their susceptibility was nonetheless reduced relative to the parental strain. These findings demonstrate that exposure to individual agricultural azoles can lead to broad-spectrum cross-resistance, affecting both other fungicides and multiple clinically relevant azoles.

**Table 1.** Antifungal susceptibility of the progenitor strain and 12 representative evolved *Candida tropicalis* strains to agricultural and medical azoles.

Antifungal agents		MIC to antifungal agents (μg/mL)												
		C23	DFC-Evo1	DFC-Evo2	DFC-Evo3	DNC-Evo1	DNC-Evo2	DNC-Evo3	FLT-Evo1	FLT-Evo2	FLT-Evo3	HXC-Evo1	HXC-Evo2	HXC-Evo3
Agri.	**DFC**	1	**32**	**32**	**32**	8	8	8	16	8	16	8	16	8
	**DNC**	0.0625	1	2	2	**4**	**4**	**4**	2	2	2	2	2	2
	**FLT**	0.25	4	8	8	4	8	8	**8**	**8**	**8**	8	8	8
	**HXC**	0.125	2	2	1	2	4	4	1	1	2	**4**	**4**	**4**
Med.	**FLC**	1	16	32	16	32	64	64	32	16	32	64	64	64
	**ITC**	1	8	8	4	32	64	64	4	4	4	64	64	64
	**KEC**	0.25	1	1	1	2	4	8	1	1	2	2	2	2
	**POS**	1	2	1	1	32	64	64	1	16	1	64	64	64
	**VRC**	0.0625	1	2	1	2	4	4	2	2	4	4	4	4

Agricultural (Agri.) azoles: DFC, difenoconazole; DNC, diniconazole; FLT, flutriafol; HXC, hexaconazole. Medical (Med.) azoles: FLC, fluconazole; ITC, itraconazole; KEC, ketoconazole; POS, posaconazole; VRC, voriconazole. Minimum inhibitory concentration (MIC) ranges (μg/mL) of azole drugs are determined following the guidelines in the Clinical and Laboratory Standards Institute (CLSI) manuals M27.

### 3.3. Morphological characteristics and growth rates of the evolved strains

Fitness trade‑offs refer to the balance or cost between competing biological traits, and such trade‑offs between growth and drug resistance are frequently observed in pathogenic *Candida* species (Vandeputte et al. 2008; Vincent et al. 2013; Carolus et al. 2021; Hu et al. 2025a). We next examined the growth phenotypes of the azole-resistant evolved strains. When cultured on YPD agar supplemented with the red dye phloxine B, the evolved strains exhibited diverse colony morphologies (Figure 2). While the progenitor strain C23 formed white colonies, most evolved strains produced pink or red colonies. Notably, HXC-evolved strains exhibited wrinkled colony morphology and higher levels of cellular aggregation. Despite these differences in colony appearance, the cellular morphologies, such as cell size and yeast-form structure, remained similar across all strains under the tested conditions.

**Figure 2.**
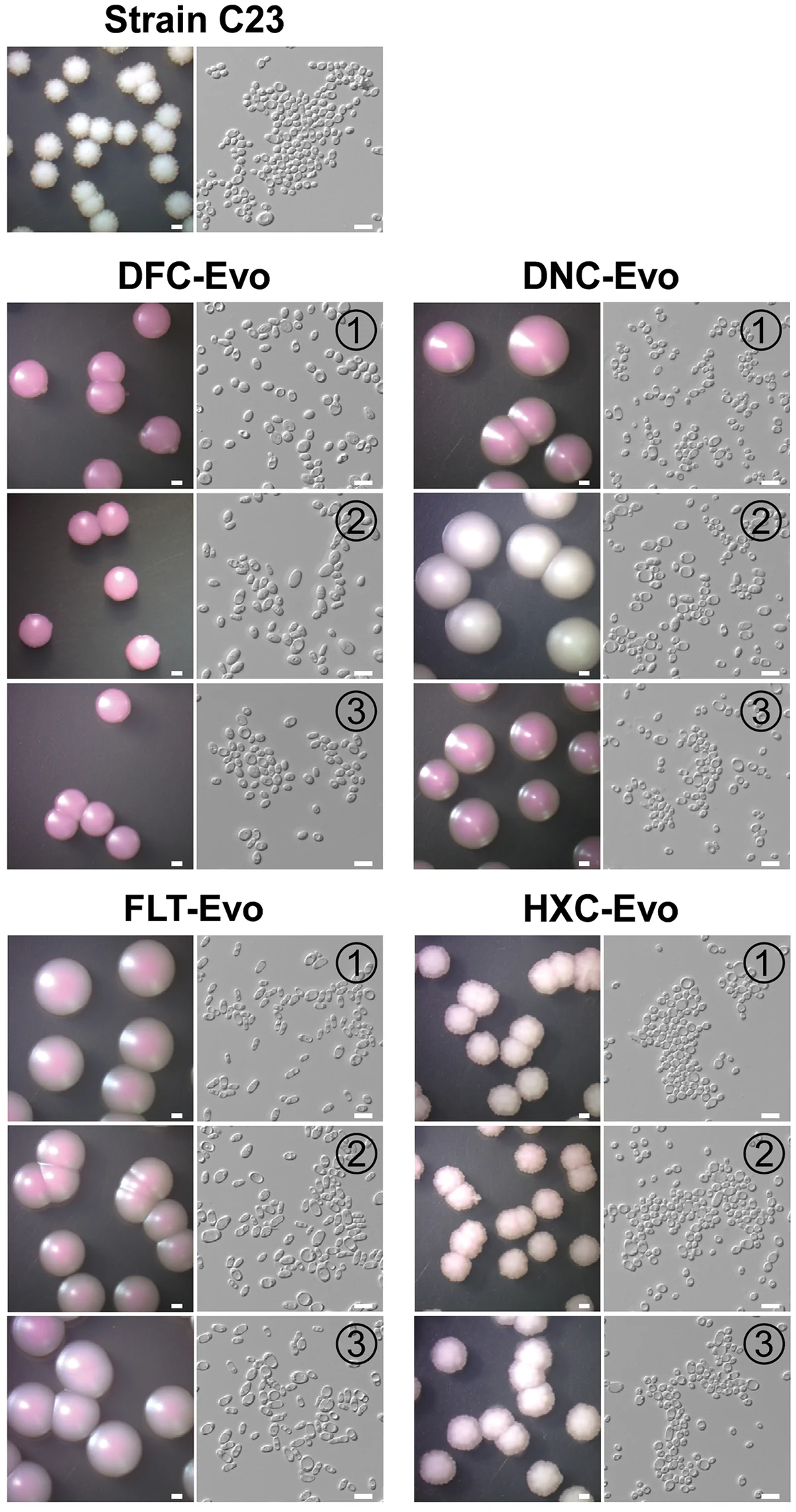
Colony and cellular morphologies of the progenitor strain and 12 representative evolved *Candida tropicalis* strains. Three evolved strains with the highest resistance level to each agricultural azole were randomly selected for morphological assays. The progenitor strain C23 served as the wild-type control (WT). Fungal cells were plated on YPD agar containing 5 μg/mL phloxine B, a red dye, and incubated at 30 °C for 3 d. Colony scale bar: 1 mm; cellular scale bar: 10 μm. 1, 2, and 3: evolved strains (Evo) Evo1, Evo2, Evo3, respectively.

Given the variation in colony size and coloration, we next assessed growth rates to evaluate potential fitness trade-offs associated with drug resistance. As shown in Figure 3, DFC- and FLT-evolved strains exhibited noticeably slower growth than the progenitor strain C23, reaching lower final cell densities (OD_600_). In contrast, two DNC-evolved strains and one HXC-evolved strain grew faster than strain C23, whereas the remaining strains exhibited slower growth. These findings indicate that a fitness trade-off associated with drug resistance occurred in only a subset of the evolved strains.

**Figure 3.**
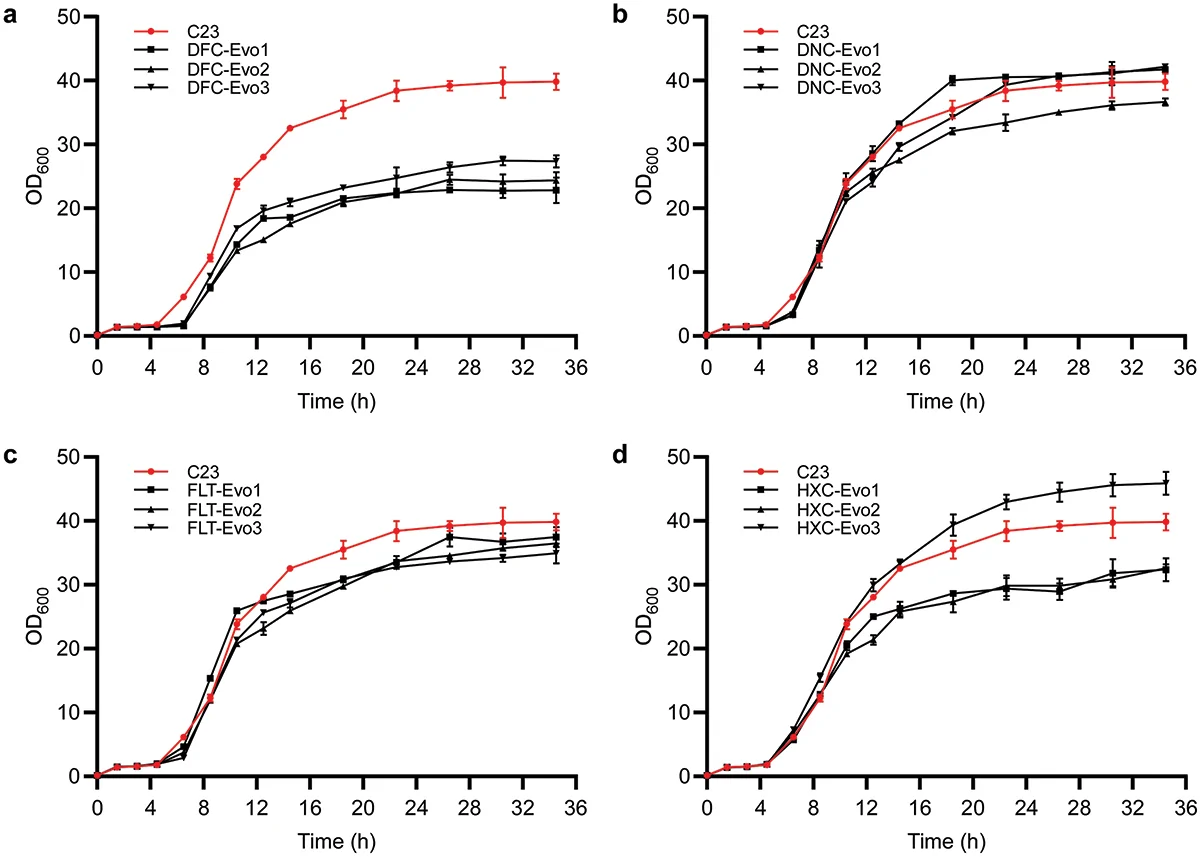
Growth curves of the progenitor strain and 12 representative evolved *Candida tropicalis* strains. (a) Growth curves of DFC-evolved strains. (b) Growth curves of DNC-evolved strains. (c) Growth curves of FLT-evolved strains. (d) Growth curves of HXC-evolved strains. Single colonies were grown overnight in liquid YPD medium at 30 °C with shaking (220 r/min), washed twice with ddH_2_O, and re-inoculated into 20 mL fresh YPD at 1 × 10^6^ cells/mL. Cultures were incubated at 30 °C with shaking (220 r/min), and growth was monitored by OD_600_ measurements at the indicated time points. Three biological repeats were performed. Evo: evolved strain.

### 3.4. Generation of aneuploidies is associated with drug resistance in the evolved strains

Because morphological variation and fitness trade-offs in pathogenic fungi are often associated with aneuploidy formation (Bing et al. 2020; Sah et al. 2021), we examined the genomic DNA content of the evolved strains by flow cytometry. As shown in Figure 4, the progenitor strain C23 exhibited a typical diploid profile, whereas all 12 evolved strains were aneuploid, with genomic DNA content ranging from 2.1 N to 2.9 N, indicating the presence of extra or segmental chromosomes.

**Figure 4.**
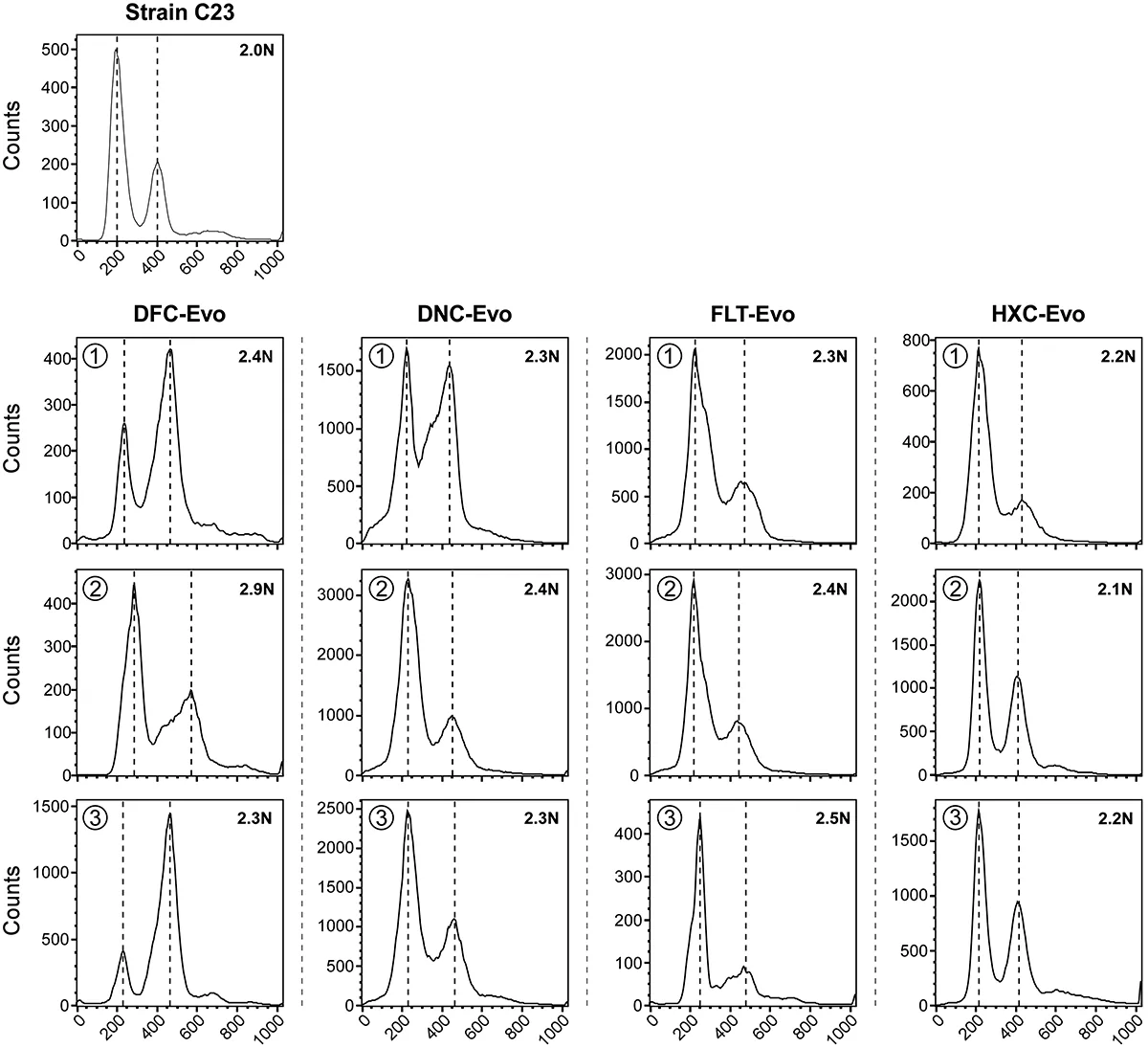
Flow cytometry analysis of the progenitor strain and 12 representative evolved *Candida tropicalis* strains. Single colonies were grown on YPD liquid medium to stationary phase, then diluted into 3 mL fresh YPD and cultured until OD_600_ = 1.8. Fungal cells were fixed, stained with propidium iodide (PI), and analyzed by flow cytometry as described in the methods. The progenitor strain C23 showed standard 2C and 4C peaks. In contrast, evolved drug-resistant *C. tropicalis* strains displayed aneuploid profiles with increased ploidy. Evo: evolved strain.

To confirm these genomic alterations, we conducted whole-genome sequencing (WGS) analysis. Compared with the diploid progenitor strain C23, all evolved strains carried either additional whole chromosomes or segmental chromosomal amplifications (Figure 5), consistent with the flow cytometry results (Figure 4). Different agricultural azoles induced distinct aneuploidy patterns, and strains evolved under the same fungicide often exhibited similar karyotypes. For example, DFC- and FLT-evolved strains shared segmental aneuploidies on chromosome 3 and whole aneuploidies of chromosome 6, whereas DNC- and HXC-evolved strains predominantly showed segmental aneuploidies on chromosome R. A summary of genomic variations is provided in Table S2. These findings suggest that agricultural azole exposure can trigger genomic instability and aneuploidy formation in *C. tropicalis*, potentially contributing to antifungal resistance, although the resistance mechanisms may vary depending on the specific azole.

**Figure 5.**
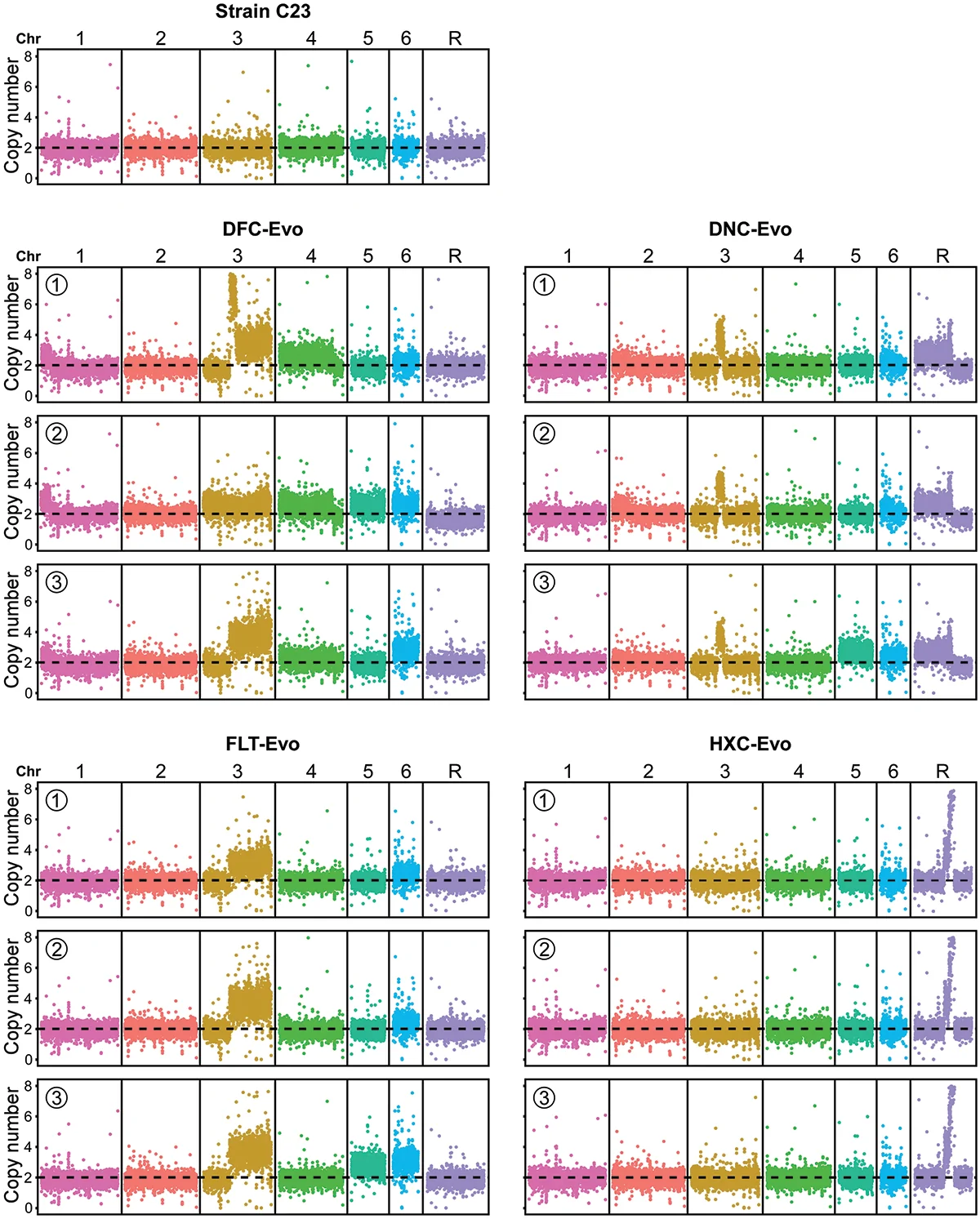
Genome-wide copy number distribution in the progenitor strain and 12 representative evolved *Candida tropicalis* strains. The x-axis represents the seven chromosomes, and the y-axis indicates relative copy number. Each point corresponds to the average copy number within a 1000-bp genomic segment based on sequencing coverage and depth. Progenitor strain: C23. 1, 2, and 3: evolved strains (Evo) Evo1, Evo2, Evo3, respectively.

We next performed comparative genomic analysis to identify patterns of aneuploidy and associated gene copy number changes. Compared with strain C23, the evolved strains carried a substantial number of genes with more than two copies, ranging from 1063 multicopy genes in FLT-Evo3 to 135 in HXC-Evo3 (Dataset S1). Many of these genes are directly or indirectly associated with drug resistance (Table S2 and Dataset S1). Notably, 9 of the 12 evolved strains carried aneuploidies of chromosome 3, which includes resistance-associated genes, such as *NPR2, NCP1, BCR1, CDR11, GZF3, IFU5, ERG25, MGE1, ERG27*, and *REP1*. Strains with additional copies of chromosome 5 contained multicopies of *CNB1*, *SNQ2*, *ERG4*, *ERG1*, *SGE1*, *TAC1*, and *FCR3*. Chromosomes 6 and R also carry genes involved in ergosterol biosynthesis and transcriptional regulation of drug resistance. These copy number variations likely contribute to the cross-resistance phenotypes observed in the evolved strains.

In addition, some evolved strains carried increased copies of genes associated with resistance to amphotericin B and caspofungin (Dataset S1). Susceptibility testing revealed that all evolved strains had reduced susceptibility to micafungin, and that FLT- and HXC-evolved strains also exhibited elevated MICs for anidulafungin (Table S3). No significant changes were detected in susceptibility to amphotericin B, caspofungin, or 5-flucytosine.

In addition to copy number variations, we indeed noted that there were some amino acid substitution mutations in drug resistance-associated genes in the evolved strains. For example, point mutations of *CDR11* were detected in DFC-Evo1, DFC-Evo2, FLT-Evo1, HXC-Evo1, and HXC-Evo2 strains, whereas mutations of *SNQ2* were found in FLT-Evo1 and FLT-Evo2 strains (Table S4). However, no evidence demonstrated that these mutations are associated with increased drug resistance in pathogenic *Candida* species in previous publications. It remains to be further investigated whether these mutations contribute to the change of drug susceptibility or have a synergistic effect on the increase of drug resistance with copy number variations or ploidy changes.

### 3.5. Global gene expression profiles in the evolved strains

To further investigate the mechanism underlying cross-resistance, we performed transcriptome analysis on the progenitor strain C23 and the 12 evolved strains. Differential expression analysis (two-fold change cut-off) identified a large number of differentially expressed genes (DEGs) in the evolved strains compared with C23 (Dataset S2). The DEG counts varied dramatically across strains, from over 1700 in FLT-Evo1 to approximately 250 in HXC-Evo1, correlating with the extent of copy number variations (CNVs) detected (Figure 5).

We identified a core set of DEGs shared among the evolved strains: 33 DEGs were differentially expressed in at least 10 strains, 27 DEGs in 11 strains, and 16 DEGs in all 12 strains (Figure 6a and Dataset S3). These shared genes were involved in antifungal resistance, stress response, virulence factor secretion, and phenotypic switching (Figure 6b,c). For example, *WOR1*, a master transcriptional regulator of white-opaque switching, was consistently upregulated across all evolved strains. *ERG1* and *ERG12*, both involved in ergosterol biosynthesis, were also upregulated. Several azole resistance-associated genes, including *ERG11*, *ERG13*, *CDR1*, and *MDR1*, showed increased transcription despite no change in copy number, suggesting that regulatory mechanisms beyond gene dosage contribute to resistance. Genes implicated in biofilm formation and filamentous growth, such as *ASR1*, *ASR2*, *YWP1*, and *CHT2*, also exhibited altered expression, potentially explaining the distinct colony morphologies observed in the evolved strains (Figures 2, 6c, and Dataset S3). Interestingly, not all genes with increased copy number were transcriptionally upregulated. For instance, *IFU5* and *CNB1* showed no significant expression change in FLT-Evo3, indicating that CNV alone does not fully determine transcriptional output.

**Figure 6.**
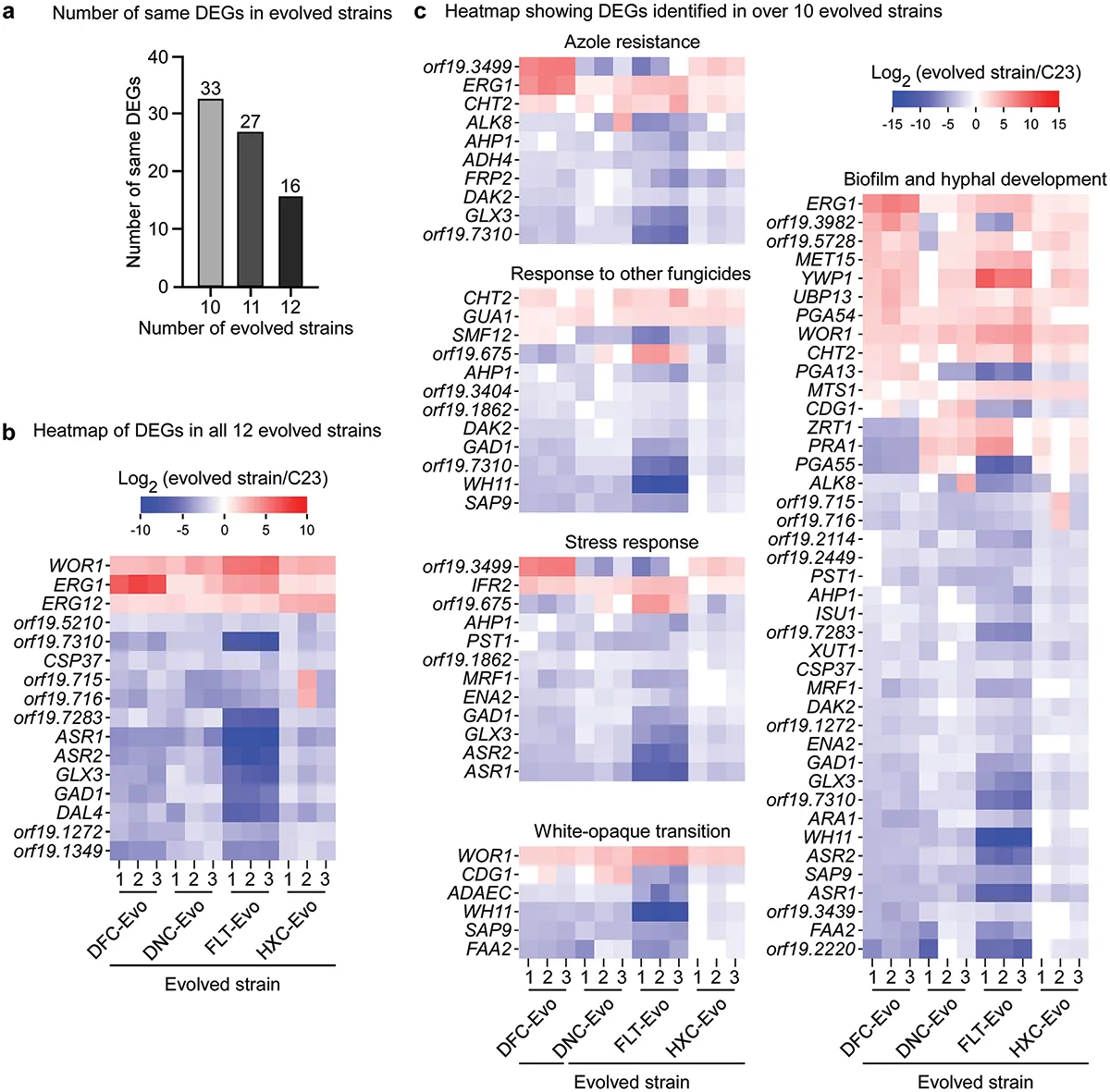
Differential expression gene (DEG) profiles between the progenitor strain and 12 representative evolved *Candida tropicalis* strains. (a) Numbers of common DEGs identified in at least 10, 11, or all 12 evolved strains. (b) Representative DEGs consistently shared across all 12 evolved strains relative to C23. (c) Representative DEGs present in at least 10 of the 12 evolved strains. DEGs were defined as genes with a ≥ 2-fold change and false discovery rate (FDR) < 0.01. Log_2_ (fold change) values are shown to indicate expression magnitude. Evo: evolved strain.

## 4. Discussion

The human fungal pathogen *C. tropicalis* is widely distributed in the environment (Opulente et al. 2019). Previous studies suggest that its reduced susceptibility to azoles may be linked to environmental exposure to agricultural fungicides (Hu et al. 2025b). In this study, we investigated whether agricultural azoles can induce cross-resistance to medical azoles in *C. tropicalis*. Using an experimental evolution approach, we exposed a susceptible strain to four commonly used agricultural azoles, including DFC, DNC, FLT, and HXC (Figure 1). The evolved strains showed markedly decreased susceptibility to both agricultural and clinical azoles. Whole-genome sequencing demonstrated that all evolved strains were aneuploid, with distinct karyotypes induced by different azoles (Figure 5). Each group of evolved strains also exhibited unique phenotypic traits and transcriptomic profiles.

Aneuploidy is frequently associated with drug resistance in pathogenic *Candida* species (Bing et al. 2020; Sah et al. 2021). For example, aneuploidy or isochromosome formation of chromosome 5 is associated with azole resistance in *C. albicans* (Selmecki et al. 2006), as this isochromosome carries genes involved in ergosterol synthesis and drug efflux. Similarly, *Candida parapsilosis* readily forms aneuploidies in response to stressors, such as tunicamycin (TUN) and aureobasidin A (AbA) (Yang et al. 2021). Azole exposure can also lead to mutations in resistance-associated genes, such as *MRR1* and *ERG3* (Papp et al. 2020; Qin et al. 2025) or to copy number increase in *RTA3* (Bergin et al. 2022). These chromosome alterations can promote drug resistance and enhance fitness under antifungal stress in *Candida* species (Hull et al. 2017).

Consistent with these findings, our results suggest that *C. tropicalis* acquires azole resistance through whole or segmental aneuploidies following agricultural azole exposure. Most evolved strains in our study carried extra copies of chromosomes 3 and R, likely reflecting the presence of resistance-related genes on these chromosomes (Figure 5 and Table S2). Despite structural similarities among agricultural azoles, each compound induced distinct chromosome alterations (Figure 5), suggesting that different azoles may trigger resistance via divergent genomic responses.

Transcriptome analysis revealed that gene expression changes were broadly consistent with the observed karyotypic alternations (Figure 5 and Dataset S2). More than 30 genes were consistently differentially expressed in at least 10 of the 12 evolved strains compared with the progenitor strain C23, indicating possible shared transcriptional responses. Notably, several drug resistance-associated genes, including *ERG11*, *CDR1*, and *MDR1*, were upregulated. We also observed differential expression of genes involved in phenotypic switching and biofilm formation, such as *WOR1*, *YWP1*, and *WH11*, which may contribute to resistance or adaptation. The precise roles of these transcriptional changes in mediating drug resistance, however, require further investigation.

It should be noted that our study still has several limitations. First, a single strain of a single species was tested in this study. Future investigations are needed to generalize the conclusion regarding the development of drug resistance in environmental fungal species. Second, there could be discrepancies between our laboratory evolutionary conditions and natural environments. The natural environments could be much more complex. The concentration level of azole fungicides could vary dramatically. Third, only a subset of naturally azole-resistant *C. tropicalis* strains exhibit gene copy number variation (Fan et al. 2023) and other mechanisms are involved in the development of drug resistance in this species. Moreover, it would be interesting to explore the effects of other non-azole fungicides on environmental pathogens.

Taken together, our findings demonstrate that *C. tropicalis* can develop cross-resistance to medical azoles after exposure to agricultural azoles. Given the widespread distribution of this pathogen in environmental niches, the role of agricultural azoles in driving resistance is of significant concern. Importantly, we found that fluconazole-resistant clinical isolates of *C. tropicalis* also exhibited reduced susceptibility to agricultural azoles. Combined with our previous observation of high azole resistance among environmental strains (Hu et al. 2023), these results suggest potential bidirectional transmission between environmental and clinical settings. Agricultural azoles may thus serve as an important environmental driver of antifungal resistance in *C. tropicalis* and other environmental fungal pathogens.

In recent years, the One Health concept, which focuses on the health connections among “humans, animals, and the environment”, has received considerable attention worldwide. To prevent the environmental emergence and transmission of azole-resistant fungal pathogens, the One Health concept should be adopted, its corresponding approaches implemented, and international collaboration strengthened. First, the misuse of azole fungicides in agriculture should be restricted and new antifungal strategies should be developed in both agriculture and clinical settings. Second, the associated policies should be made and applied to strengthen the harmless treatment of agricultural sewage and breeding waste. Third, the application of antifungal food preservatives should be limited.

## Supplementary Material

Supplemental Material
