# Supplementary material for: Agricultural azole fungicides induce cross-resistance to medical antifungals in Candida tropicalis: a One Health perspective on antifungal resistance
Source: Mycology. 2026 Apr 23;17(3):972–85. doi: 10.1080/21501203.2026.2657142 (PMC13647410; doi:10.1080/21501203.2026.2657142)
Supplement: Supplementary file 1 [file TMYC_A_2657142_SM6203.zip › Supplementary file-0409.docx]

**Supplementary files**

**Table S1. Strains used in this study.**

| **Strain name** | **Parent strain** | **Description** | **Reference** |
| --- | --- | --- | --- |
| C23 |  | Commensal isolate | Hu et al. (2023) |
| DFC-Evo1 | C23 | Evolved by difenoconazole | This study |
| DFC-Evo2 | C23 | Evolved by difenoconazole | This study |
| DFC-Evo3 | C23 | Evolved by difenoconazole | This study |
| DNC-Evo1 | C23 | Evolved by diniconazole | This study |
| DNC-Evo2 | C23 | Evolved by diniconazole | This study |
| DNC-Evo3 | C23 | Evolved by diniconazole | This study |
| FLT-Evo1 | C23 | Evolved by flutriafol | This study |
| FLT-Evo2 | C23 | Evolved by flutriafol | This study |
| FLT-Evo3 | C23 | Evolved by flutriafol | This study |
| HXC-Evo1 | C23 | Evolved by hexaconazole | This study |
| HXC-Evo2 | C23 | Evolved by hexaconazole | This study |
| HXC-Evo3 | C23 | Evolved by hexaconazole | This study |

**Reference:**

Hu T, Wang S, Bing J, Zheng Q, Du H, Li C, Guan Z, Bai F, Nobile C, Chu H, et al. 2023. Hotspot mutations and genomic expansion of *ERG11* are major mechanisms of azole resistance in environmental and human commensal isolates of *Candida tropicalis*. Int J Antimicrob Agents. 62(6):107010. doi: 10.1016/j.ijantimicag.2023.107010.

**Table S2. Location of potential azole-resistant genes on chromosomal segments with copy number changes.**

| **Strains** | **Chromosome No.** | **Sequence location** | **Average fold-change** | **Involved genes with copy number change*** |
| --- | --- | --- | --- | --- |
| **DFC-Evo1** | 1 | 2−342 kb | 1.5× | Not observed |
|  | 3 | 953−1208 kb | 3× | *NPR2* (7)*, NCP1* (7) |
|  | 3 | 1209−2502 kb | 2× | *BCR1* (3)*, CDR11* (3)*, GZF3* (3)*, IFU5* (3)*, ERG25* (3)*,*  *MGE1* (3)*, ERG27* (3)*, REP1* (3) |
|  | 4 | 2−1973 kb | 1.5× | *MDR1* (3) |
| **DFC-Evo2** | 1 | 2−342 kb | 1.5× | Not observed |
|  | 3 | 1−2502 kb | 1.5× | Not observed |
|  | 4 | 2−1973 kb | 1.5× | Not observed |
|  | 5 | 1−1239 kb | 1.5× | *CNB1* (3)*, SGE1* (3)*, TAC1* (3) |
|  | 6 | 2−925 kb | 1.5× | Not observed |
| **DFC-Evo3** | 1 | 2−342 kb | 1.5× | Not observed |
|  | 3 | 953−2502 kb | 2× | *NPR2* (4)*, NCP1* (3)*, BCR1* (4)*, CDR11* (3)*, GZF3* (4)*,*  *IFU5* (4)*, ERG25* (3)*, MGE1* (3)*, ERG27* (3)*, REP1* (4) |
|  | 6 | 2−925 kb | 1.5× | *YCF1* (3)*, GRP2* (3)*, HSP90* (3)*, FLU1* (3) |
| **DNC-Evo1** | 3 | 953−1208 kb | 2× | *NPR2* (4)*, NCP1* (4) |
|  | R | 9−1358 kb | 1.5× | *ERG13* (3)*, orf19.6382* (3)*,*  *ALK8* (3)*, STT4* (3)*, TPO3* (3)*, MIR1* (3) |
| **DNC-Evo2** | 3 | 953−1208 kb | 2× | *NPR2* (4)*, NCP1* (4) |
|  | R | 9−1358 kb | 1.5× | *ALK8* (3)*, UPC2* (3) |
| **DNC-Evo3** | 3 | 953-1208 kb | 2× | *NPR2* (4)*, NCP1* (4) |
|  | 5 | 1−1239 kb | 1.5× | *CNB1* (3)*, SNQ2* (3)*, ERG4* (3)*, ERG1* (3)*,*  *SGE1* (3)*, TAC1* (3)*, FCR3* (3) |
|  | R | 9−1358 kb | 1.5× | *ALK8* (3)*, STT4* (3)*, UPC2* (3)*, ERG12* (3) |
| **FLT-Evo1** | 3 | 953−2502 kb | 1.5× | *NPR2* (3)*, NCP1* (3)*, BCR1* (3)*, CDR11* (3)*, GZF3* (3)*,*  *IFU5* (3)*, ERG25* (3)*, ERG27* (3)*, REP1* (3) |
| **FLT-Evo2** | 3 | 953−2502 kb | 1.5× | *NPR2* (4)*, NCP1* (4)*, BCR1* (3)*, CDR11* (4)*, GZF3* (4)*,*  *IFU5* (3)*, ERG25* (4)*, MGE1* (4)*, ERG27* (3)*, REP1* (3) |
| **FLT-Evo3** | 3 | 953−2502 kb | 1.5× | *NPR2* (3)*, NCP1* (4)*, BCR1* (3)*, CDR11* (3)*, GZF3* (4)*,*  *IFU5* (4)*, ERG25* (3)*, MGE1* (3)*, ERG27* (3)*, REP1* (3) |
|  | 5 | 1−1239 kb | 1.5× | *CNB1* (3)*, SNQ2* (3)*, ERG4* (3)*,*  *ERG1* (3)*, SGE1* (3)*, FCR3* (3) |
|  | 6 | 2−925 kb | 1.5× | *YCF1* (3)*, GRP2* (3)*, HSP90* (3)*, FLU1* (3)*, ERG5* (3) |
| **HXC-Evo1** | R | 1084−1464 kb | 3× | *MIR1* (4)*, RAM2* (8)*, ERG12* (9)*, PXA2* (10) |
| **HXC-Evo2** | R | 1084−1464 kb | 3× | *MIR1* (4)*, RAM2* (8)*, ERG12* (9)*, PXA2* (10) |
| **HXC-Evo3** | R | 1084−1464 kb | 3× | *MIR1* (4)*, RAM2* (8)*, ERG12* (9)*, PXA2* (9) |

* Copy numbers are shown in the brackets.

**Table S3. Antifungal susceptibility of the progenitor strain and 12 representative evolved *Candida tropicalis* strains to non-azole drugs.**

| **Antifungal**  **agents** | **MIC to antifungals（μg/mL）** | | | | | | | | | | | | |  |
| --- | --- | --- | --- | --- | --- | --- | --- | --- | --- | --- | --- | --- | --- | --- |
|  | Strain C23 | DFC-Evo1 | DFC-Evo2 | DFC-Evo3 | DNC-Evo1 | DNC-Evo2 | DNC-Evo3 | FLT-Evo1 | FLT-Evo2 | FLT-Evo3 | HXC-Evo1 | HXC-Evo2 | HXC-Evo3 | |
| **5-FC** | 0.125 | 0.25 | 0.25 | 0.25 | 0.25 | 0.25 | 0.25 | 0.25 | 0.5 | 0.25 | 0.25 | 0.5 | 0.25 | |
| **AmB** | 2 | 2 | 2 | 2 | 2 | 2 | 2 | 2 | 4 | 2 | 2 | 2 | 2 | |
| **AFG** | 0.5 | 1 | 1 | 1 | 1 | 1 | 1 | 4 | 4 | 4 | 4 | 2 | 1 | |
| **CAS** | 0.125 | 0.25 | 0.125 | 0.25 | 0.25 | 0.25 | 0.5 | 0.5 | 1 | 0.5 | 0.25 | 0.5 | 0.25 | |
| **MFG** | 0.125 | 1 | 1 | 1 | 1 | 0.5 | 0.5 | 1 | 1 | 1 | 0.5 | 1 | 1 | |

Antifungals: 5-FC, 5-flucytosine; AmB, amphotericin B; AFG, anidulafungin; CAS, caspofungin, MFG, micafungin. Minimum inhibitory concentration (MIC) ranges (μg/mL) of antifungal drugs are determined following the guidelines in the Clinical and Laboratory Standards Institute (CLSI) manuals M27. Evo: evolved strain.

**Table S4. Detection of azole resistance-related gene mutations in evolved strains.**

| **Strain** | **Azole resistance related gene** | **Amino acid mutations** |
| --- | --- | --- |
| **DFC-Evo1** | *ERG251* | S49del |
|  | *CDR11* | K421Q |
|  | *CDR2* | T982A |
|  | *orf19.6382* | F1229L |
|  | *STT4* | H239R, D1439A, E1596K |
|  | *MLT1* | T665A |
| **DFC-Evo2** | *ERG251* | S49del |
|  | *CDR11* | K421Q |
|  | *orf19.6382* | F1229L |
|  | *STT4* | H239R, D1439A, E1596K |
|  | *MLT1* | F113S, T665A |
| **DFC-Evo3** | *MRR2* | D613E |
|  | *ERG251* | S49del |
|  | *orf19.6382* | F1229L |
|  | *STT4* | H239R, D1439A, E1596K |
|  | *MLT1* | F113S, T665A |
| **DNC-Evo1** | *CDR2* | T982A |
|  | *MLT1* | T665A |
| **DNC-Evo2** | *MRR2* | D613E |
|  | *CDR2* | V15M, Y274S |
|  | *CRZ1* | I231M |
|  | *MRR1* | T87A |
|  | *MLT1* | L622F, T665A |
| **DNC-Evo3** | *MRR2* | D613E |
|  | *CDR2* | V15M, Y274S |
|  | *CRZ1* | I231M |
|  | *MRR1* | T87A |
|  | *MLT1* | L622F, T665A |
| **FLT-Evo1** | *CDR11* | K421Q |
|  | *SNQ2* | D791G |
|  | *ERG4* | Q6R |
|  | *TAC1* | I164N, S278L, S448Y |
|  | *MLT1* | F113S, T665A |
|  | *CDR1* | F1077L |
| **FLT-Evo2** | *SNQ2* | D791G |
|  | *ERG4* | Q6R |
|  | *TAC1* | I164N, S278L, S448Y |
|  | *MLT1* | F113S, T665A |
| **FLT-Evo3** | *MRR2* | D613E |
|  | *TAC1* | S448Y |
|  | *MLT1* | F113S, T665A |
| **HXC-Evo1** | *CDR11* | K421Q |
| **HXC-Evo2** | *CDR11* | K421Q |
|  | *MLT1* | T665A |
| **HXC-Evo3** | Not observed | Not observed |

* A total of 62 azole resistance-related genes, including *ALK8, BCR1, CAP1, CDR1, CDR11, CDR2, CDR3, CNB1, CRZ1, CYB5, ERG1, ERG10, ERG11, ERG12, ERG13, ERG2, ERG24, ERG25, ERG251, ERG26, ERG27, ERG28, ERG3, ERG4, ERG5, ERG6, ERG9, FCR3, FLU1, GAL1, GZF3, GRP2, HSP90, HXK2, IFU5, MDR1, MGE1, MIR1, MLT1, MRR1, MRR2, MSI3, NCP1, NPR2, orf19.6382, PDR16, PDR17, PIR1, PXA2, RAM2, REP1, ROA1, RTA2, SGE1, SNQ2, STT4, TAC1, TPO3, UPC2, VPS28, YCF1,* and *YOR1*, were subjected to mutation detection.

**S1_Dataset: Categories of drug-related genes with copy number variations.**

**S2_Dataset: Categories of differentially expressed genes associated with azole resistance.**

**S3_Dataset: Categories of differentially expressed genes in at least 10 of the 12 evolved strains.**
